# Postmenopausal Women With Osteoporosis and Musculoskeletal Status: A Comparative Cross-Sectional Study

**DOI:** 10.4021/jocmr537w

**Published:** 2011-07-26

**Authors:** Sylvia Cunha-Henriques, Lucia Costa-Paiva, Aarao Mendes Pinto-Neto, Gislaine Fonsechi-Carvesan, Livio Nanni, Sirlei Siani Morais

**Affiliations:** a Department of Obstetrics and Gynecology, School of Medical Sciences, Universidade Estadual de Campinas (UNICAMP), Brazil

## Abstract

**Background:**

With increased life expectancy of the world's population that has taken place in recent decades, there has been growth in the incidence of illnesses of the most advanced ages, including osteoporosis. However, changes in musculoskeletal disorders are not yet so clear. This study proposes to evaluate musculoskeletal alterations in osteoporotic postmenopausal women and healthy and correlate with bone mineral density of the lumbar spine.

**Methods:**

Randomized, examiner-blinded, comparative cross-sectional study was designed with two groups of women attending the Menopause Clinic in the UNICAMP, 30 women with osteoporosis, while 33 women without osteoporosis comprised the second group. Diagnosis of the presence or absence of osteoporosis was based on bone densitometry performed on the lumbar spine. Volunteers were interviewed and underwent a physical examination with the same examiner, including the muscle strength and amplitude of movement of back flexion and extension, angles of thoracic kyphosis and lumbar lordosis, as well as static and dynamic balance.

**Results:**

Mean back flexors and extensors strength was significantly lower in women with osteoporosis (P < 0.01). Flexion spinal range of motion was similar in both groups (P = 0.91). However, movement amplitude of spine extension was 20.5^o^ in women with osteoporosis and 28.4^o^ in women without osteoporosis. Thoracic kyphosis angles from T1 to T4 (P < 0.01) and lumbar lordosis angles (P = 0.02) were greater in women with osteoporosis. Seventy-three point three percent of women with osteoporosis and 78.8% of women without osteoporosis had good reply to static balance. Women in both groups had poor results to dynamic balance. No significant differences were observed in static or dynamic balance between women with and without osteoporosis. Vertebral fractures were present in 20% of women with osteoporosis and absent in women without osteoporosis.

**Conclusions:**

Women with osteoporosis in the study population had poorer musculoskeletal status than women without osteoporosis. Further studies are necessary to evaluate whether correction of these alterations would be related to preventing falls and reducing fracture risk.

**Keywords:**

Balance; Kyphosis; Mobility; Muscle strength; Osteoporosis; Postmenopausal

## Introduction

Osteoporosis is a progressive disease that has physical and psychosocial consequences [1]. It has already been well established that when bones become weaker, muscle status changes, causing modifications to posture and increasing the probability of falls and fracture, since the center of gravity is modified, leading to a loss of body balance [2]. Some studies have shown a reduction in muscle strength in the dorsolumbar region of women with osteoporosis, and have reported a correlation between this condition and bone mineral density in the lumbar spine, possibly aggravating postural abnormalities associated with the disease [3-6]. In general, disorders related to postural curvature of the spine, such as in thoracic kyphosis and lumbar lordosis, are present in those patients with osteoporosis who have suffered greater loss of stature [7-10].

In addition to the normal sagittal alignment of the spine and adequate muscle strength, flexibility and balance are also necessary to combat the effects of gravity, as well as other external forces. A reduction in range of motion and deterioration in coordination that affects body balance is a consequence of osteoporosis as well as advanced age itself [11].

Bearing in mind that the presence of osteoporosis in postmenopausal women may be associated with musculoskeletal disorders and that these disorders are considered risk factors for the occurrence of falls and fractures, techniques need to be developed to diagnose these disorders in postmenopausal women with osteoporosis through the use of readily available and inexpensive instruments. The research objectives were to evaluate musculoskeletal disorders (muscle strength, range of motion of the spine, kyphosis and lordosis index, and balance) in healthy and osteoporotic postmenopausal women, and correlate these variables with bone mineral density of the lumbar spine.

## Materials and Methods

A randomized, examiner-blinded, comparative cross-sectional study was conducted.

This study was carried out in 63 women with and without osteoporosis, who were followed up at the menopause clinic of Comprehensive Health Care of Women Center in State University of Campinas (CAISM/UNICAMP), Brazil. White women aged 40 - 65 years, who had been in amenorrhea for at least 12 months and who had been submitted to bone densitometry within the six months prior to admission, were included in the study. Women who had used sedatives or tranquilizers in the 30 days preceding admission or those who had any neurological disease or any disorder that affected balance, any musculoskeletal disease with deformity of the lower limbs, or malignant neoplasia of any type were excluded from the study.

No statistically significant differences were observed between the two groups in mean age, education, age at last menstruation and time since menopause.

The study was carried out by a single examiner who was the physiotherapist responsible for the research. The radiographs were requested by the examiner, performed and reports were completed by a professor at the Department of Radiology, Faculty of Medical Sciences at UNICAMP.

The women was selected sequentially in agreement order of admission in the clinic at the moment of the medical consultation, since that they filled the necessary criteria for inclusion in the study.

The study was approved by the Internal Review Board of the Department of Gynecology and Obstetrics, School of Medical Sciences, and the Research Ethics Committee of the School of Medical Sciences, (blinded). All participants signed an informed consent form prior to admission. The protocol is designed to conform to the principles of the Declaration of Helsinki.

### Intervention

Two groups were formed consisting of 30 women with osteoporosis and 33 who did not have osteoporosis. Diagnosis was made using bone densitometry of the lumbar spine (segment L2-L4), according to the densitometry criteria defined by the World Health Organization [12].

An initial interview was carried out by the examiner (a physiotherapist) during which information was collected with respect to age, education, age at menopause, time since menopause, use of hormone replacement therapy, practice of physical activity, and frequency and duration of any physical activity performed, as well as measurement of weight and height for the calculation of body mass index. The evaluator did not know which group belonged to volunteer.

### Outcome measures

#### Muscle strength

Back extensors strength had been evaluated by the Manual Muscle Test (MMT) (simple method, known and trained for the physiotherapists in general) with the patient in the ventral decubitus while back flexors strength in the supine position. Function of the primary muscle group was observed three times by the examiner (bigger value was used) in order to assess the full-amplitude of movement. According to Daniels' criteria [13], the muscle strength was graded from 0 (absence muscular contraction) to 5 (able to move part of the body against gravity and against an additional resistance).

#### Range of motion

**Figure 1.**
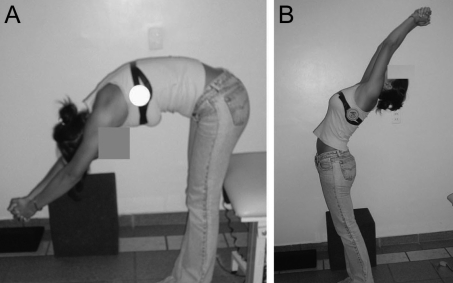
Range of motion of the spine, flexion (A) and extension (B), measured with fleximeter®.

The range of motion of the total spine was measured in the upright position and at maximum flexion/extension using a Fleximeter® (www.institutocode.com) while the subject maintained joined inferior members, extended knees and parallel feet, and extended arms and interlaced fingers. On the lateral face of the thorax, fleximeter® was placed at the height of the armpits and was requested it flexion or the extension of the trunk once each movement. The maximum limit achieved by the movements was expressed in degrees (Fig. 1).

#### Kyphosis index and lumbar lordosis index

Sagittal curvature of the spine was evaluated using simple profile radiography of the thoracic and lumbar spine (way of relatively easy access), applying the method established by Cobb for obtaining the desired kyphosis and lordosis angles [9]. The kyphosis angle was evaluated between the first (T1), second (T2), third (T3) or fourth (T4) and the twelfth (T12) thoracic vertebrae, and the lordosis angle between the first lumbar vertebra (L1) and the first sacral vertebra (S1). A ruler was used to trace a line along the upper edge of the first visible thoracic vertebra, and another line was drawn along the lower edge of the T12 vertebra. A line was then drawn perpendicular to these two lines. The point of congruence gave the kyphosis angle, which was measured using a goniometer. The same procedure was carried out to evaluate the lordosis angle between vertebrae L1 and S1. All radiographies were analyzed by a radiologist who was blinded to the subject's original group.

#### Postural balance [14]: static

The measurement of the ability of the patient to maintain the center of gravity on the support base while in a standing position was performed using the Romberg test (test very indicated in similar studies) which was carried out by requesting the subject to remain barefoot, in a standing position, with her head held high, her arms crossed in front of her body and her eyes open. After establishing the correct position, the subject was requested to close her eyes and the time during which she managed to maintain this position was then measured in seconds, using a chronometer that had been tested for 60 seconds. Positive was considered when the volunteer lost the balance before completing the 60 seconds of the test.

#### Postural balance: dynamic

The ability of the patient to maintain the center of gravity on the base of support at the center of two circles (58 and 70 cm each) was measured through the Unterberger stepping tests, while the subject moved her lower limbs, simulating walking on the spot, with her eyes open, and then closing her eyes for 60 seconds. This test was evaluated using the same position of Romberg test and graded according to the patient's ability as: 3 (subject uses only the smaller support area), 2 (subject uses the smaller and medium support areas), and 1 (patient exceeds the two support areas).

#### Vertebral fracture

This was defined either as a reduction of more than 20% in anterior height of the larger vertebra in relation to posterior height or adjacent vertebra, or vertebral collapse, identified from lateral X-ray radiographs view of the dorsolumbar spine, and recorded as either present or absent [2].

#### Bone mineral density

Measured using Dual Energy X-Ray Absorptiometry (DEXA) of the lumbar spine (segment L2-L4), using a LUNAR DPX bone densitometer (LUNAR Corporation), according to WHO criteria [12]. Coefficient of variation was 1%.

### Data analysis

A descriptive statistical analysis was first carried out using frequency, mean and standard deviation. For the comparison of frequencies, the chi-square test or Fisher's exact test was used, and for the comparison of continuous variables, Mann-Whitney's non-parametric test was applied. To evaluate the association between sagittal curvatures of the vertebral column, muscle strength, range of motion of the spine (flexion/extension), static and dynamic balance, and bone mineral density, Spearman's rank correlation test was used. To evaluate variables associated with L2-L4 bone mineral density, multiple linear regression was used, and results were adjusted for body mass index (BMI), age, use of hormone therapy, physical activity and age at last menstruation. A difference of P < 0.05 was considered statistically significant. The SAS statistical software package, version 8.2, was used for all statistical analyses.

## Results

Sixty-three women were recruited (2 groups: with and without osteoporosis) and underwent some musculoskeletal tests. The characteristics of subjects in both study groups are summarized in Table 1.

**Table 1:** Clinical Characteristics of the Postmenopausal Women With and Without Osteoporosis

Variable	Women with Osteoporosis (n = 30) (mean ± SD)	Women without Osteoporosis (n = 33) (mean ± SD)	P-value*
Age (years)	57.40 ± 5.21	55.76 ± 5.76	0.35
Education (years of schooling)	3.70 ± 4.36	5.24 ± 4.33	0.09
Age at menopause (years)	46.70 ± 7.15	45.33 ± 5.94	0.23
Time since menopause (years)	11.00 ± 6.65	11.06 ± 6.53	0.97
Weight (kg)	57.70 ± 13.64	71.34 ± 13.57	< 0.01
Height (cm)	142.29 ± 32.62	150.23 ± 27.43	0.01
BMI (kg/m^2^)	25.62 ± 6.34	29.74 ± 5.90	< 0.01

* Mann-Whitney non-parametric test

Body mass index was significantly different in the group of women with osteoporosis (25.62 ± 6.34) compared to the group of women without osteoporosis (29.74 ± 5.9), (P < 0.01). Concerning to the use of hormone replacement therapy, 66.7% of the group with osteoporosis and 69.7% of the group without osteoporosis were using hormone therapy and there was no statistically significant difference between the two groups.

The mean grade of back flexor strength in the group of women with osteoporosis was significantly lower than that of the women without osteoporosis (P < 0.01). The group of women with osteoporosis showed half the strength in the back extensor muscle when compared with the women without osteoporosis, and this difference was statistically significant (P < 0.01).

There were no statistically significant differences in the mean range of flexion movement of the spine between those women with osteoporosis and those who did not have osteoporosis; however, the mean range of extension movement of the spine was significantly less (P < 0.01) in the group of women with osteoporosis (Table 2).

**Table 2:** Mean Grade of Muscle Strength and Range of Motion of the Spine (Flexion/Extension) in Postmenopausal Women With and Without Osteoporosis

Variable	Women with osteoporosis (n = 30) (mean ± SD)	Women without osteoporosis (n = 33) (mean ± SD)	P-value*
Muscular strength			
Trunk flexors	1.43 ± 0.50	2.00 ± 0.79	< 0.01
Trunk extensors	1.10 ± 0.48	2.09 ± 0.98	< 0.01
Range of motion			
Spine flexion	88.17 ± 13.74	86.67 ± 16.85	0.91
Spine extension	20.50 ± 9.68	28.48 ± 10.12	< 0.01

* Mann-Whitney non-parametric test

The measurements of the thoracic T1 to T4 kyphosis angles varied from 60.31^o^ to 62.10^o^ in the women with osteoporosis and from 47.00^o^ to 49.82^o^ in the women without osteoporosis. Comparison of the thoracic kyphotic angles revealed significantly greater measurements in the women with osteoporosis at all vertebra evaluated (P < 0.01). With respect to the lordosis angle, a statistically significant difference was also seen between the two groups, the women with osteoporosis having significantly higher values compared to the women without osteoporosis (P < 0.01) (Table 3).

**Table 3:** Mean Thoracic Kyphosis and Lumbar Lordosis Angles in Postmenopausal Women With and Without Osteoporosis

Angles of the sagittal curvatures of the spine		Women with osteoporosis		Women without osteoporosis		P-value*
		n	Mean ± SD	n	Mean ± SD	
Thoracic kyphosis angle	T1	18	60.94 ± 4.80	17	49.82 ± 3.83	< 0.01
	T2	26	60.77 ± 4.94	23	47.39 ± 5.87	< 0.01
	T3	29	60.31 ± 5.13	33	47.64 ± 6.49	< 0.01
	T4	30	62.10 ± 5.64	33	47.00 ± 6.70	< 0.01
Lumbar lordosis angle		30	117.60 ± 8.09	33	53.12 ± 5.15	< 0.01

* Mann-Whitney non-parametric test

With respect to the static balance test, the women in both groups had good reply, that is, negative Romberg test (73.7% of the women with osteoporosis versus 78.8% of the women without osteoporosis) but bad response to dynamic balance for exceeding the circles demarcated in the soil (78.8% of the women with osteoporosis versus 86.7% of the women without osteoporosis). There was no statistically significant difference between the two groups (P = 0.48). When chronometer the time that each volunteer obtained to remain itself in balance, the mean durations of static (52.4 versus 52.5 seconds; P = 0.99) and dynamic balance (25.6 versus 27.0 seconds; P = 0.61) were similar in the two groups (Table 4).

**Table 4:** Static and Dynamic Balance of Postmenopausal Women With and Without Osteoporosis

Balance			Women with osteoporosis (n = 30)	Women without osteoporosis (n = 33)	P-value
Static	Grade (%)	negative	73.3	78.8	0.61**
		positive	26.7	21.2	
	Time (mean)		52.43 ± 15.50	52.58 ± 15.92	0.99***
Dynamic	Grade (%)	3	9.1	10.0	0.48*
		2	12.1	3.3	
		1	78.8	86.7	
	Time (mean)		27.00 ± 18.28	25.60 ± 19.30	0.61***

* Fisher's exact test; ** Chi-square test; *** Mann-Whitney non-parametric test

**Figure 2.**
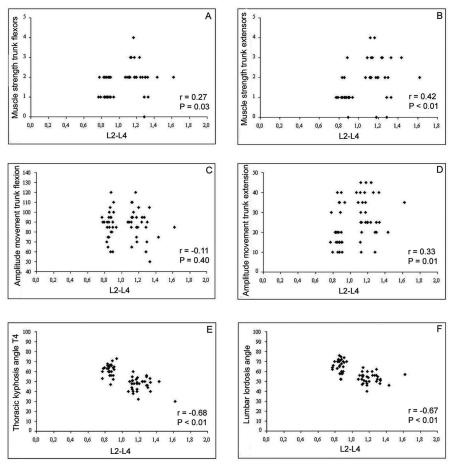
Correlation between bone mineral density of lumbar spine L2-L4 and muscle strength of trunk flexors (A), trunk extensors (B), amplitude of movement trunk flexors (C), trunk extensors (D), thoracic kyphosis angle T4 (E), and lumbar lordosis angle (F).

A significant direct correlation was observed between flexor and extensor muscle strength and bone mineral density (g/cm^2^) in the L2-L4 segment. The higher the bone mineral density, the greater the muscle strength, the degree of correlation being greater in the case of the back extensor (r = 0.42; P < 0.01) (Fig. 2A, B). A significant direct correlation was also noted between the amplitude of extension movement and bone mineral density (r = 0.33; P = 0.01), while the amplitude of flexion movement showed no correlation (P = 0.04) (Fig. 2C, D). Correlation analysis revealed an inverse association between the thoracic T1 to T4 kyphosis angles (r = -0.64, -0.60, -0.66, -0.68, respectively; P < 0.01), the lumbar lordosis angle (r = -0.67; P < 0.01) and L2-L4 bone mineral density. The lower bone mineral density, the greater thoracic kyphosis and lumbar lordosis angles (Fig. 2E, F).

Multiple linear regression analysis for BMI, age, use of hormone therapy, physical activity and age at menopause showed an association between the presence of osteoporosis and lower back flexion-extension strength, lower range of extension movement of the spine and greater angles of T4 kyphosis (Table 5).

**Table 5:** Factors Associated With Bone Mineral Density of L2-L4 (g/cm^2^) According to Multiple Linear Regression Analysis

Variable	Estimated parameter	Coefficient error	R^2^	P-value*
Range of motion: extension spine	0.0036	0.0020	0.143	0.03
T4 kyphosis angle	-0.0125	0.0019	0.603	< 0.01

* Adjusted for BMI, age, use of HT, physical activity and age at menopause.

## Discussion

This study compared musculoskeletal alterations in postmenopausal women with or without osteoporosis. One of the alterations observed in the participants studied was a reduction in muscle strength. Back flexor and extensor strength was poorer in women with osteoporosis. This finding is in agreement with data published by other authors showing a significant correlation between reduced back muscle strength and a decrease in the bone mineral density of the vertebral column [15].

In 1993, Sinaki et al [7] compared extensor muscle strength of the spine of postmenopausal women with and without osteoporosis. These investigators reported that strength was reduced in women with osteoporosis and that this reduction increased with age. These data show that there are reductions in extensor muscle strength and a presence of deformities of the skeleton such as kyphosis and other postural abnormalities associated with osteoporosis. For this reason, exercises to strengthen the back extensors may be important in the management of deformities related to osteoporosis [16].

Sinaki et al [17] carried out a randomized study that included 10 years of follow-up of postmenopausal women submitted to an intensive exercise program to strengthen the back extensor muscles. These authors reported an increase in bone mineral density in the spine of women participating in the exercise program, and demonstrated a fold lower relative risk of compression fracture in this group as compared to the control group.

It is important to emphasize that mean measurements of back flexor and extensor strength were indicative of deficiencies in the two groups of women evaluated, the majority obtaining a grade below 3, which is considered the minimal normal grade. This is explained by the average age of the women in this study, which is over 45 years of age, since deteriorations in musculoskeletal conditions are common in this age group. This is due not only to deterioration as a result of inadequate postural habits but also to the inactivity that is often seen in women in this age group. Nevertheless, it was possible to confirm the obvious loss of muscle strength in the group of postmenopausal women in whom this reduction was aggravated by the presence of osteoporosis.

The Epidemiologic Study on the Prevalence of Osteoporosis in Italy showed that sarcopenia is considered to be one of the main features of the aging process. It is characterized by a reduction in muscle mass and muscle strength, and affects women more than men. It is associated with an increased risk of fractures consequent upon a greater predisposition to falls, but also to the lack of bone remodeling due to reduced muscle mechanical strength. Muscle strength determines the quality of bone modifications such as density, strength, and microarchitecture. Variations in the ratios of cortical and muscle areas give rise to various types of osteoporosis, with different risks of fracture [4].

No significant differences were seen between the two groups with respect to the range of flexion motion of the spine, possibly related to exacerbation of the kyphosis angle, favoring forward curvature of the spine in the women with osteoporosis, or perhaps for possible low degree of flexibility of the hamstring muscles in the two studied groups. However, the values found for range of extension motion of the spine were lower in the group of women with osteoporosis, confirming data from other studies. According to literature women with osteoporosis had reduced flexibility and mobility that affected their walking and contributed towards a greater risk of falling and fracture, as well as frequent episodes of pain and less independence of movement, all of these conditions being aggravated when associated with an inactive lifestyle. Diminished range of motion may also be caused by muscular resistance or by muscular contractures often present in osteoporosis patients, and frequently found in osteoporotic patients with spine deformities such as thoracic kyphosis [18, 19].

Hyperkyphotic posture is a well-known complication of osteoporosis [5]. The results found about thoracic kyphosis vary according to the level of the vertebrae evaluated and according to the location, type and number of vertebral fractures [9]. Thoracic kyphosis angle was evaluated using simple lateral radiograph, and significantly greater angles were observed in women with osteoporosis. Some authors have reported that, in the age group studied, the weakness of the back extensor muscles and the reduction in the elasticity of the intervertebral discs, in addition to the action of gravity and the patient's lifestyle, may result in kyphotic posture even when osteoporosis is not present [20]. This fact should be taken into account. Thoracic kyphosis is a risk factor for vertebral fractures over 3 years, and influences physical capacity changes, in postmenopausal women with osteoporosis [21]. Several studies have shown that the increase in kyphosis angles and vertebral deformities may lead to reduced physical mobility and a lower quality of life, lower self-esteem, a reduced capacity to carry out daily activities, and may result in episodes of both acute and chronic pain [9, 22].

Mean thoracic kyphosis and lumbar lordosis angles were greater in women with osteoporosis. Since the back is inclined further forward because of the kyphosis, the levering arm movement is therefore greater and added to the action of the force of gravity; these factors contribute towards making the extension movement more difficult. If we associate this factor with reduced back flexor and back extensor strength, we may conclude that one fact justifies the other. In other words, this muscular imbalance (weakness in the back extensor muscles) may also favor the aggravation of the sagittal curvatures of the vertebral spine and limit range of motion.

No statistically significant differences were found with respect to balance between women with and without osteoporosis; nevertheless, it is important to emphasize that dynamic balance was bad in 86% of the women with osteoporosis and 78% of the women without osteoporosis. Although difference in the balance between the groups was not observed, it fits to comment the important option for evaluating the balance, as well as the too much alterations, through tests readily available and inexpensive, that they supply graduations less precise than tests of balances dynamic with equipment with measured quantitative direct. Other studies have shown that women with osteoporosis also present alterations of balance, measured through these dynamic tests [23]. Perhaps the present study could show more important alterations in balance in women with osteoporosis, if test with direct measures was used. However, it bears mentioning that the test of Romberg is widely used, having great clinical applicability that allows measuring the activities of daily life in similar situations and is low cost.

Studies have shown that there are differences in the strategies used to control balance and postural instability in women with and without osteoporosis. Lynn et al [11] reported that osteoporotic women used greater compensation strategies in the hip region than at the ankles to maintain balance than women without osteoporosis. In women with accentuated thoracic kyphosis, greater postural instability has also been reported [24].

Most studies relate changes in balance to advanced age and the presence of fractures, particularly of the hip [11, 25]. In the present study, only a few women had fractures, which may be a possible explanation for the differences observed.

Although the results of this study fail to show any differences in balance due to osteoporosis, our finding of poor balance in postmenopausal women is a relevant fact that may be explained by the influence of age. The differences in the strategies of balance between young and elderly women have been reported by various authors, who observe that older women have reduced lower limb muscle strength and poorer sensorial-motor efficiency [11, 26].

Some authors show different results of this study in relation to the curvature of lumbar spine, where lumbar (not thoracic) kyphosis and spinal inclination have a statistical correlation with postural sway. Postural deformity with lumber kyphosis may represent as a risk factor for falls [27].

One possible limitation of this study is that it is a cross-sectional study in which the interpretation of the cause-effect association between osteoporosis and musculoskeletal disorders may have been affected by the fact that measurement of the events was not carried out in the logical sequence in which they occurred. Moreover, the musculoskeletal alterations studied may have been influenced by factors other than the presence of low bone mass. The fact that we have used non-objective quantification tests of muscle strength and balance may have led to an under-detection of the more subtle changes in these parameters, contributing towards a lower validity of the measurements carried out. Nevertheless, the practical tests applied in this study are commonly used and have been validated in the literature.

In conclusion, this study presents that women in this age group with osteoporosis may suffer deterioration in musculoskeletal status that in the long term may result in limitations to their daily activities, as well as lumbar pain, an increase in the occurrence of falls, and a consequent increase in fracture risk. Minimally invasive, low cost and easily applied techniques can be used to identify women with osteoporosis, who should then be counseled to begin exercise programs to promote and increase strength and flexibility. Functional recovery of women with osteoporosis would minimize the consequences of the disease. It should be emphasized that population studies are of vital importance in identifying risk factors for falls and fractures in the different populations in order to help physicians who provide healthcare to postmenopausal women.

Prospective studies should be carried out to evaluate whether physiotherapy would be effective in minimizing these alterations so that programs of functional recuperation can be implemented to reduce these risks, particularly in the case of patients with osteoporosis.
